# Effect of febuxostat on renal function in patients from South China with CKD3 diabetic nephropathy

**DOI:** 10.1590/2175-8239-JBN-2019-0091

**Published:** 2020-07-22

**Authors:** Huang Wen, Zhu Yongling, Zheng Shuying, Wang Jiali, Zhao Yanling

**Affiliations:** 1 Wenzhou Medical University, Second Affiliated Hospital of Wenzhou Medical University, Department of Nephrology, Wenzhou, China.; 2 Wenzhou City Second People's Hospital, Hematological Department, Wenzhou City, China.

**Keywords:** Febuxostat, Renal Insufficiency, Chronic, Diabetic Nephropathies, Hyperuricemia, Febuxostat, Insuficiência Renal Crônica, Nefropatias Diabéticas, Hiperuricemia

## Abstract

**Objective:**

To investigate the efficacy and safety of febuxostat on renal function in CKD stage 3 diabetic nephropathy patients.

**Methods:**

Patients in our hospital with chronic kidney disease (CKD) stage 3 diabetic nephropathy (DN) complicated by high serum uric acid (360 µmol/L) were recruited. Patients were then divided into treatment group and control group according to the random number table method. All the patients received low purine diet, renin-angiotensin-aldosterone system (RAAS) inhibitors, and adequate routine hypoglycemic treatment. Febuxostat was employed only in the treatment group. The levels of blood uric acid (sUA), serum creatinine (Scr), cystatin C (cys-c), eGFR, 24-hour urine protein quantification, albuminuria, and creatinine ratio (ACR) were evaluated in all patients before and after treatment at 4, 8, 12, and 24 week.

**Results:**

No difference was found before treatment between the two groups. After treatment at 4, 8, 12, and 24 week, the levels of sUA, SCr, cys-c, and eGFR between the two groups were significant different (P<0.05). There was no difference in 24-hour urine protein quantification, albuminuria, and creatinine ratio between two groups before treatment, and significant differences were observed after treatment. Fifty percent of patients from the treatment group achieved the treatment goal with 20 mg febuxostat at 4 weeks. Tubular markers were also decreased with the treatment.

**Conclusions:**

Febuxostat can reduce uric acid and improve renal function effectively in patients with CKD stage 3 diabetic nephropathy, while being well tolerated. However, the conclusion is still uncertain due to the short term of the study.

## RESUMO

**Objetivo:**

Investigar a eficácia e segurança do febuxostat na função renal em pacientes com DRC estágio 3, com nefropatia diabética.

**Métodos:**

Foram recrutados pacientes em nosso hospital com nefropatia diabética (DN) estágio 3 de doença renal crônica (DRC) complicada por ácido úrico sérico alto (360 µmol/L). Os pacientes foram então divididos em grupo de tratamento e grupo controle, de acordo com o método da tabela de números aleatórios. Todos os pacientes receberam dieta pobre em purinas, inibidores do sistema renina-angiotensina-aldosterona (RAAS) e tratamento hipoglicêmico de rotina. O Febuxostat foi empregado apenas no grupo de tratamento. Os níveis de ácido úrico no sangue (AIU), creatinina sérica (Scr), cistatina C (cys-c), TFGe, quantificação de proteínas na urina em 24 horas, razão albumina e creatinina (ACR) foram avaliados em todos os pacientes antes e após o tratamento às 4, 8, 12 e 24 semanas.

**Resultados:**

Nenhuma diferença foi encontrada antes do tratamento entre os dois grupos. Após o tratamento nas 4, 8, 12 e 24 semanas, os níveis de sUA, SCr, cys-c e TFGe entre os dois grupos foram significativamente diferentes (P <0,05). Não houve diferença na quantificação de proteínas na urina em 24 horas, albuminúria e razão de creatinina entre dois grupos antes do tratamento, e diferenças significativas foram observadas após o tratamento. Cinquenta por cento dos pacientes do grupo de tratamento atingiram a meta de tratamento com 20 mg de febuxostat em 4 semanas. Marcadores tubulares também foram reduzidos com o tratamento.

**Conclusões:**

O Febuxostat pode reduzir o ácido úrico e melhorar a função renal efetivamente em pacientes com nefropatia diabética estágio com DRC no estágio 3, sendo bem tolerado. No entanto, a conclusão ainda é incerta devido ao curto prazo do estudo.

## INTRODUCTION

Diabetic nephropathy is the leading cause of renal failure, of which morbidity is still very high. According to recent surveys^1^, diabetic nephropathy now is the most common cause of hospitalization of chronic renal failure. Data from nearly 20 years of research in the United States show the prevalence of end stage kidney disease caused by diabetic nephropathy does not decreased even after controlling the complications of diabetes, such as cardiovascular and cerebrovascular events^2^. The treatment of diabetic nephropathy is still the dilemma confronting the nephrologist.

Uric acid treatment in diabetic nephropathy patients has been a focus of nephrology for a long time. Initially, there is a reciprocal effect between elevation of uric acid and diabetic nephropathy, then a vicious cycle ensues, as evidenced by some research^3^. Epidemiological investigations and animal research show that hyperuricemia (HUA) poses an independent risk for chronic kidney disease in the setting of diabetes^4^.

Febuxostat, approved by the United States FDA (Food and Drug Administration) in 2009 and successfully listed as a selective xanthine oxidase inhibitor, is now widely used in gout patients as a uric acid-reducing drug. Compared with other uric acid-lowering drugs, febuxostat is less renal toxic when used in renal insufficiency patients. There are some reports on the efficacy and safety of febuxostat in CKD patients^5^^,^ 6. However, there are very few reports on the clinical efficacy and safety of febuxostat in type 2 DN patients complicated with hyperuricemia^7^^,^ 8. In this study, febuxostat was used to treat patients with CKD3 diabetic nephropathy in order to explore its efficacy and safety.

## MATERIALS AND METHODS

### CRITERIA FOR INCLUSION, EXCLUSION AND DROP OUT

Inclusion criteria were age from 18 to 70 years, in line with the diagnostic criteria of DN according to the guidelines of prevention and control of type 2 diabetes mellitus of China (2013 edition), eGFR ranging from 59 to 30 mL/min/1.73m^2^ according to the 2012 CKD-EPI equation (Chronic Kidney Disease Epidemiology Collaboration (CKD-EPI) equation): eGFR=130×(SCr/0.7)^‒0.601^×(Cys C/0.8)^‒0.711^×0.995^age^ (female, SCr > 0.7, Cys C > 0.8), eGFR=135×(SCr/0.9)^‒0.601^×(Cys C/0.8)^‒0.711^×0.995^age^ (male, SCr > 0.9, Cys C > 0.8), units of eGFR, SCr, Cys C are mL/(min·1.73m^2^), mg/dL, mg/L, respectively), serum uric acid greater than 6 mg/mL (360µmol/L).

Exclusion criteria were body mass index greater than 28 kg/m^2^, past history of gout attacks, hbA1c higher than 7%, systolic pressure greater than 140 mmHg, diastolic pressure greater than 90 mmHg, abnormal liver function, white blood cell less than 3×10^9^/L or/and hemoglobin less than 80 g/L or/and platelet count less than 100×10^9^/L or more than 300×10^9^/L, chronic active hepatitis B or hepatitis B surface antigen (HBsAg) positive or/and hepatitis Be antigens (HBeAg) positive, hepatitis C virus infection, tuberculosis, HIV infection, severe fungal infections, or other severe uncontrolled infections, tumor history, allergy to febuxostat, previous administration of mercaptopurine hydrate, azathioprine, cytarabine, use of losartan, fenofibrate, thiazide diuretics, medullary loop diuretics, allopurinol, benzene bromide, febuxostat, probenecid, within the last 4 weeks, acute renal injury, blood creatinine fluctuation greater than 50% in the previous 3 months before enrollment, alcohol and drug abuse, and pregnancy and lactation. An informed consent was signed by patients who agreed to participate in the trial.

Drop-out criteria were participants who failed to follow the research program, or who could not finish the research according to the research program, participants who quitted the research, elevation of ALT, AST, or bilirubin to 2 times the upper normal limit for 2 weeks during the use of febuxostat, increase of blood creatinine greater than 50% of the base value, allergy, pregnancy, patients who were intolerant of gastrointestinal discomfort, unexplained serious complications, and missing person.

### SUBJECTS

Forty-two type 2 diabetes patients with diabetic nephropathy and hyperuricemia hospitalized in the Second Affiliated Hospital of Wenzhou Medical University from January 2017 to May 2018 were recruited. Patients were divided into treatment group (20 cases) and non-treatment group (22 cases) with the random number table method. There was no significant difference in the distribution of patients’ according to gender and age (p>0.05) (see Table 1). This research program was approved by the hospital’s Medical Ethics Committee, all patients signed the informed consent letter.

**Table 1:** Basic information of treatment group and control group

	Treatment Group (n=18)	Non-treatment group (n=20)
Sex (M/F)	16/2	17/3
Age (years)	58.73±11.50	57.46±10.96
Body mass index (kg/m2)	24.34±2.59	24.05±3.15
Systolic pressure (mmHg)	129.88±6.48	130.04±6.43
Diastolic pressure (mmHg)	76.42±4.70	75.03±4.75
Diabetes course (years)	11.78±5.71	12.10±5.69
Diabetic nephropathy course of disease (years)	3.64±1.53	3.71±0.65
Diabetic retinopathy (with/without)	10/8	11/9
Diabetic peripheral neuropathy (with/without)	9/9	9/11

### TREATMENT

All patients received low purine diet, RAAS inhibitors, and adequate routine hypoglycemic treatment. Febuxostat was used in patients from the treatment group (Hangzhou Zhu Yangxin Pharmaceutical Co., Ltd., license permission number: Sinopharm Z19993147, 40 mg per pill). The initial dose was 20 mg/d, 1 time daily, increasing the dose to 40 mg/d 4 weeks later if the serum uric acid was not less than 6 mg/mL (360 µmol/L). The dose was increase to 60 mg/d at 8 week if the serum uric acid was still higher than 6 mg/mL (360 µmol/L). Treatment duration was six months, and the dose of 60 mg/d was continued if the serum uric acid was not up to the standard. For patients whose serum uric acid was less than 2 mg/dL for 4 weeks, the dose was reduced to the previous level. Participants were withdrawn from the study if serum uric acid was less than 2 mg/dL when taking febuxostat at the dose of 20 mg/d. Uric acid reduction treatment was not used in the control group.

### MARKERS AND TESTING METHODS

The levels of sUA, SCr, serum statin C, 24-hour urinary protein excretion, ACR, and eGFR were evaluated before and after treatment at 0, 4, 8, 12, and 24 weeks in all patients. Intravenous blood was collected, and the ADVIA2400 automatic biochemical instrument (Chemistry System) and chemiluminescence method were used to determine sUA and SCr. The latex enhanced immune turbidity method was used to determine serum cystatin c level, the urine protein was quantitatively measured with immune scattering turbidity method, and the urinary creatinine was tested by enzymatic method. eGFR was calculated according to the 2012 CKD-EPI formula (recommended by KDIGO - Kidney Disease: Improving Global Outcomes, KDIGO - guidelines in 2012 and ADA (American Diabetes Association)guidelines in 2014). Liver function, lipids, serum glucose, blood route test were performed at 0, 4, 8, 12, and 24 weeks on all the patients from both groups. The occurrence of symptoms was recorded in all patients, including itchy skin, gastrointestinal discomfort, joint pain, muscle pain, mental abnormalities, rash, heart and brain accidents and other rare discomfort reactions (see Diagram S1).

### STATISTICAL ANALYSIS

SPSS 18.0 software was used for statistical analysis, measurement data was reported as means±SD. The difference between two groups was compared by two independent sample t-test for continuous variables. Counting data was reported as number or rate, and χ^2^ test was used for comparison. Two-way ANOVA for repeated measurements was used to test the differences of eGFR, uric acid between those two groups over 24 weeks. P<0.05 was considered a significant difference.

## RESULTS

### LOST CASES

Two patients from the treatment group quitted the research, one with respiratory tract infection within one month of treatment and one with gastrointestinal discomfort (the researchers judged that this symptom may be related to therapeutic drugs); eventually, 18 cases completed the study. In the non-treatment group, two patients lost a visit, and finally twenty cases completed the study.

### GENERAL CONDITIONS

There was no statistical difference in sex, age, body mass index, basic blood pressure, diabetes mellitus and diabetic nephropathy, diabetic complications between treatment and non-treatment groups (p>0.05); see Table 1.

### LABORATORY TESTS

**Figure 1:**
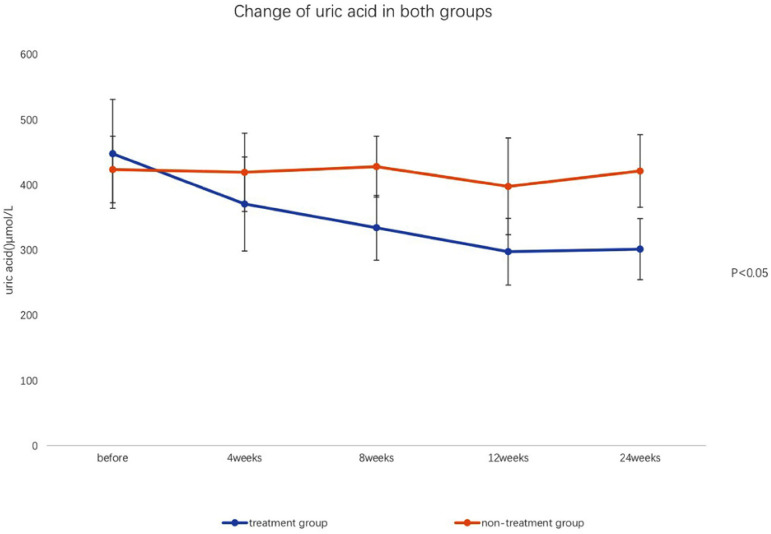
Changes of uric acid in both groups over time.

**Figure 2:**
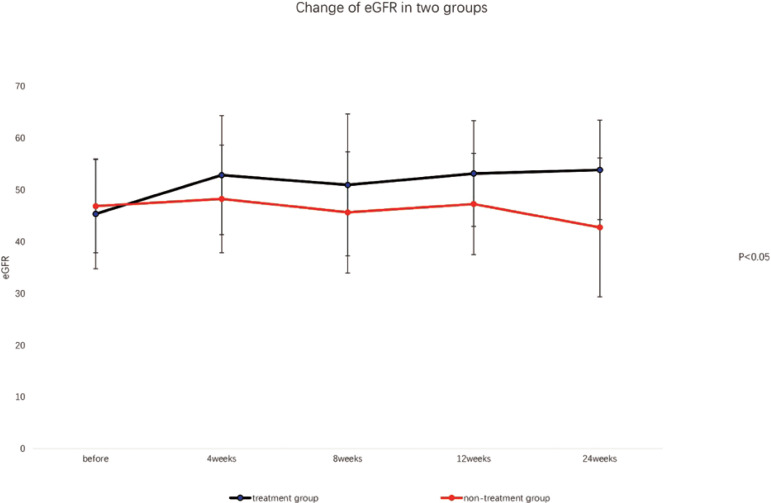
Changes of eGFR in both groups over time.

After the treatment with 20 mg febuxostat daily for 4 weeks, the serum uric acid levels of nine patients in the treatment group (50%) were below 360 µmol/L; serum uric acid levels of five cases in treatment group were below 360 µmol/L with added dose of 40 mg daily febuxostat at 8 weeks. All the patients achieved the targeted goal (serum uric acid levels below 360 µmol/L). The average dose of febuxostat was 33.4 mg. There was no significant difference in sUA, SCr, cys-c, eGFR, 24-hour urinary protein, and ACR levels between the two groups before the treatment (P>0.05). However, there were significant differences in those markers between groups at 4, 8, 12, and 24 weeks after the treatment (P<0.05). Also, those markers were significant different in different time-points in the treatment group (P<0.05), as shown in Table 2, Table 3, Figure 1, and Figure 2.

**Table 2:** sUA, SCr, cys-c, eGFR levels before and after treatment (means±SD).

Group	n	time	sUA (µmol/L)	SCr (µmol/L)	cys-c (mg/L)	eGFR (mL/min)
Treatment group	18					
before treatment			447.5±83.6	172.9±20.1	1.98 ±0.21	45.3±10.6
treatment for four weeks			370.5±72.1* ▲	141.1±24.9* ▲	1.35 ±0.16* ▲	52.8±11.5* ▲
eight weeks			334.1±49.8* ▲	139.8±35.1* ▲	1.28 ±0.13* ▲	50.9±13.7* ▲
twelve weeks			297.4±51.1* ▲	132.9±27.8* ▲	1.30 ±0.22* ▲	53.1±10.2* ▲
twenty-four weeks			301.2±46.9* ▲	148.1±30.2* ▲	1.33±0.33* ▲	53.8±9.6* ▲
non-treatment group	20					
before treatment			423.4±51.2	157.7±38.3	1.55±0.291	46.8±9.0
treatment for four weeks			419.1±60.1	163.2±35.9	1.48±0.40	48.2±10.4
eight weeks			427.8±46.8	153.3±29.8	1.53±0.33	45.6±11.7
twelve weeks			397.4±74.2	169.9±40.3	1.61±0.29	47.2±9.8
twenty-four weeks			421.1±55.7	170.6±51.9	1.70±0.52	42.7±13.4

* Significant difference between the treatment group and the non-treatment group (P<0.05);

▲ significant differences before and after treatment in the treatment group (P<0.05).

**Table 3:** Comparison of 24 h urine protein, ACR, urinary n-acetyl-β-d-(NAG)/creatinine (Cr), urinary β2 globulin (B2-MG)/creatinine, urinary α1 globulin (A1-MG)/creatinine between treatment and non- treatment groups, and before and after treatment (means±SD).

Time	Group	24h urine protein (g/L)	ACR (mg/mmol)	urine NAG/Cr (U/mmol)	Urine β2-MG/Cr (mg/mmol)	Urine α1-MG/Cr (mg/mmol)
Before treatment	Treatment	4.15±2.58	439.62±79.10	3.75±0.92	159.32±61.25	4.26±0.54
	Non-treatment	4.02±2.67	441.06±78.52	3.54±0.83	161.19±62.01	4.31±0.52
Treatment at four weeks	Treatment	3.17±2.65* △	331.56±76.45* △	2.71±0.89* △	131.12±60.93* △	3.18±0.49* △
	Non-treatment	4.10±2.58	441.45±78.68	3.59±0.82	161.42±62.19	4.37±0.50
Eight weeks	Treatment group	2.85±0.89* △	201.06±78.82* △	1.65±0.90* △	98.22±60.41* △	1.97±0.51* △
	Non-treatment	4.21±2.66	442.96±79.04	3.63±1.01	162.05±61.97	4.45±0.53
Twelve weeks	Treatment	2.80±1.59* △	191.06±92.12* △	1.71±0.46* △	106.01±80.01* △	1.83±0.61* △
	Non-treatment	4.81±2.09	501.32±79.65	3.90±1.81	222.05±49.31	5.12±1.09
Twenty four weeks	Treatment	3.07±1.21* △	191.06±92.12* △	2.32±1.06* △	126.01±60.98* △	2.08±0.30* △
	Non-treatment	4.26±2.45	491.65±70.99	4.58±1.11	282.71±99.43	4.92±2.41

* Significant difference between treatment and non-treatment groups (P<0.05);

△ significant differences before and after treatment in the treatment group (P<0.05).

### ADVERSE REACTIONS

No adverse reactions occurred in both groups, such as cardiovascular events, severe allergic reactions, etc. The results are shown in Table 4.

**Table 4:** Comparison of adverse reaction in treatment and non-treatment groups. Data are reported as n of cases (%).

Group	N	Leucopenia	Lesão hepática	Náusea	Infecção	Eritema	Coceira	Ataque agudo de gota
Treatment group	18	1(5)	2(10)	2(10)	2(10)	1(5)	3(15)	0
Non-treatment group	20	2(10)	3(15)	1(5)	2 (10)	1(5)	1(5)	0

P>0.05 between groups.

## DISCUSSION

Elevated serum UA is an important risk in the development of DN, which has been confirmed by a research showing that the risk for annual decrease of GFR of 3 ml·min^-1^·(1.73m^2^)^-1^ increased by 14% for every elevation of serum uric acid by 88.4 µmol/L^9^. Gout patients with CKD should be treated with uric acid reduction drugs 4 to 8 weeks after the control of acute gout attack until uric acid is less than 6 mg/dL (360 µmol/L), unless intolerant or adverse reactions occurred^10^. Febuxostat, another type of drug that can inhibit uric acid production, was recommended as a first-line drug by ACR (American College of Rheumatology) guideline in 2012. Several studies have shown that renal insufficiency is a risk factor for severe skin allergic reactions induced by allopurinol, especially in the Chinese Han population^11^. Therefore, diabetic nephropathy patients with serum uric acid level more than 360 µmol/L were selected in this experiment.

In recent years, studies (including clinical studies) have shown that high uric acid can cause renal damage, hypertension, and cardiovascular disease, which prompt us to treat high uric acid patients with CKD to delay the progress of renal function. However, we still look forward to better research with larger samples, higher quality, and longer duration in order to obtain more conclusive evidence. In our research, we confirmed that febuxostat could reduce the level of serum uric acid in all patients with CKD3 diabetic nephropathy, and the targeted value of serum uric acid could be easily achieved, with no severe adverse events. The research confirmed the efficacy and safety of febuxostat in CKD3 diabetic nephropathy patients.

Uric acid reduction therapy can slow down the progression of renal function in patients with CKD. Uric acid in patients and experimental animals can directly lead to microvascular lesions in the afferent arterioles, causing vascular endothelial cell damage, accelerating the progression of DN^12^. Uric acid can increase the expression of renin, then change the hemodynamics of glomerular filtration through angiotensin II, inducing systemic hypertension and glomerular hypertension as well as renal interstitial tubular injury, finally resulting in glomerular sclerosis and interstitial fibrosis^13^^,^ 14. A recent research by Hong^15^ showed that hyperuricemia can induce mitochondrial calcium overload mediated by Na^+^/Ca^2+^ exchanger, which can cause endothelial dysfunction. Hyperuricemia can also promote the secretion of cytokines like tumor necrosis factor alpha, transformation growth factor β1 and mononuclear cell chemokines-1, etc., resulting in an inflammatory cascade reaction, which in turn causes kidney damage^16^. In the settings of diabetes mellitus, various transferal disorders of metabolic substrate lead to abnormal synthesis of ATP, which affect the normal function of renal tubular epithelial cells, and then renal tubular injury and fibrosis ensue^17^. Our experiment showed the levels of SCr, Cys-C, and eGFR were significantly different in the treatment group after 4, 8, 12, and 24 weeks of treatment, and compared to that of before treatment, which showed that lowering uric acid could improve the renal function and delay the progress of renal failure in patients with CKD stage 3 diabetic nephropathy.

Through uric acid reduction treatment, the levels of α1-MG, urinary NAG, and β2-mg in urine, which can reflect the renal tubular injury, were all reduced. It is suggested that by reducing the release of cytokines and blocking the effect of inflammatory cascade reaction, lowering uric acid might mitigate the mitochondrial dysfunction of renal tubules caused by hyperglycemia and high uric acid^16^. Further clinical and animal experiments are needed to confirm this hypothesis. In our experiment, it was observed that with uric acid reduction, the high urinary NAG, β2-MG, and β2-MG levels were reversed with febuxostat, suggesting that the renal tubular damage can be alleviated with the decrease of uric acid.

Albuminuria can predict the degree of renal dysfunction in DN, which is of great significance for monitoring the curative effect of diabetic nephropathy. Annayya R showed the amount of urinary protein in 2DM patients was positively correlated with serum uric acid in his study^18^. All patients had proteinuria in this experiment, and there was no significant difference in urinary proteins between the two groups before treatment. After uric acid reduction treatment, the amount of proteinuria was significantly decreased. Those results indicated that reduction of uric acid can decrease the proteinuria in CKD stage 3 diabetic nephropathy patients, thus protect the renal function, which is consistent with previous studies.

At present, guidelines by ACR, EULAR (European League Against Rheumatism), and APLAR (Asia-Pacific League of Associations for Rheumatology) do not recommend reduction of uric acid in asymptomatic hyperuricemia patients due to the lack of prospective randomized controlled trials. It is controversial whether there is a need to reduce uric acid in asymptomatic hyperuricemia patients; the exact time to treat it or the exact drug to use are unknown. Our results supported aggressive treatment if serum uric acid was higher than 360 µmol/L. However, due to the limited sample size, short observation time, single research center, and not having a placebo group, our collusions are not so reliable.

## CONCLUSION

Febuxostat can safely reduce uric acid and improve renal function effectively in patients with CKD stage 3 diabetic nephropathy. Multi-center, clinical research with a large sample size is needed to further confirm the results.

## SUPPLEMENTARY MATERIAL

The following online material is available for this article:

**Diagram S1:** Effect of febuxostat on renal function in patients with CKD 3 diabetic nephropathy.
