# Supplementary material for: Effect of febuxostat on renal function in patients from South China with CKD3 diabetic nephropathy
Source: J Bras Nefrol. 2020 Jul 22;42(4):393–9. doi: 10.1590/2175-8239-JBN-2019-0091 (PMC7860659; doi:10.1590/2175-8239-JBN-2019-0091)

Braz. J. Nephrol. (J. Bras. Nefrol.)
https://doi.org/10.1590/2175-8239-JBN-2019-0091

Supplementary Material to: "Effect of febuxostat on renal function in patients from South China with CKD3 diabetic nephropathy"

Diagram S1 - Effect of febuxostat on renal function in patients with CKD3 diabetic nephropathy.

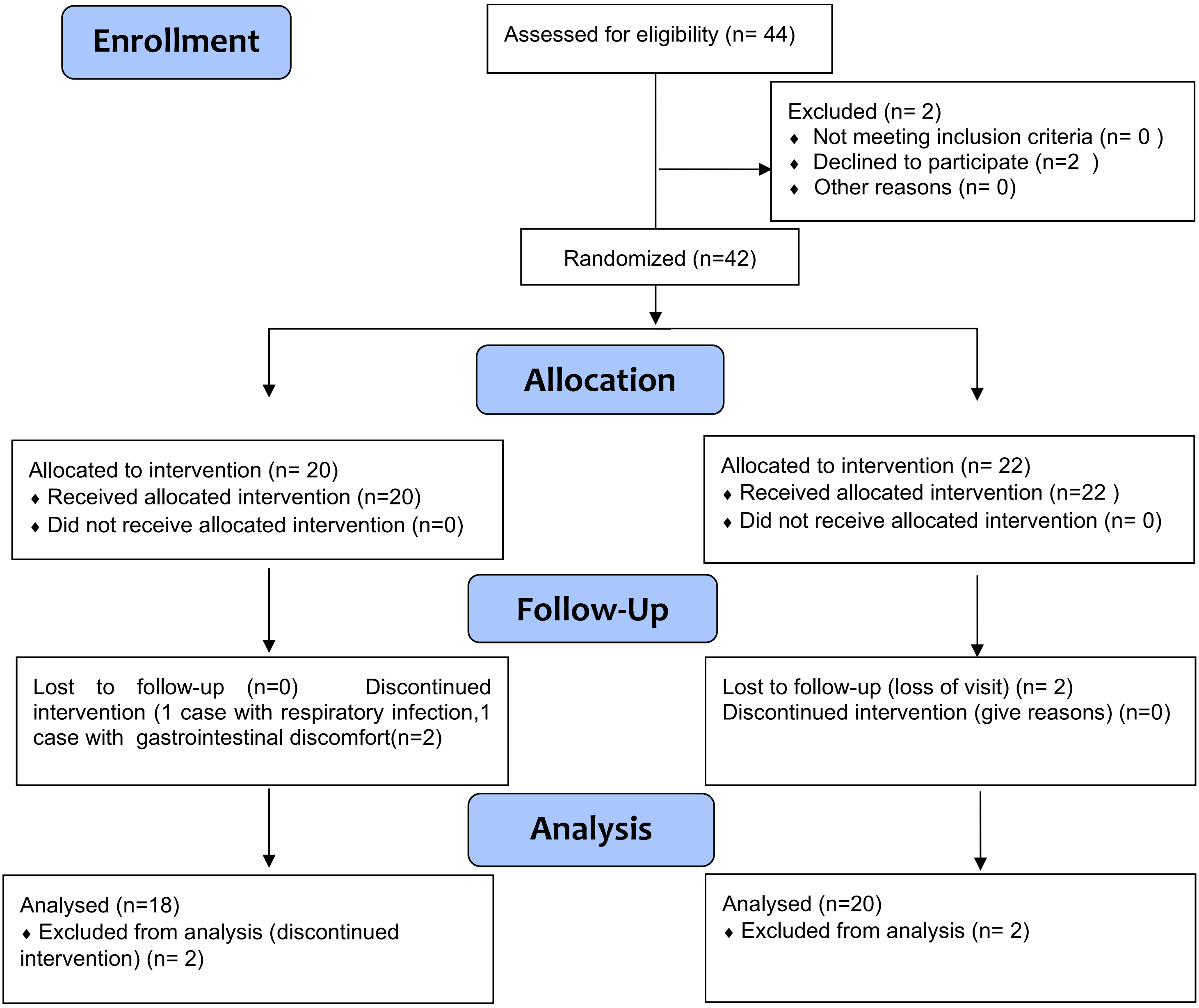

Supplement: Supplementary file 1 [file 2175-8239-jbn-2019-0091-suppl01.pdf]
